# Transient Tear Film Dysfunction after Cataract Surgery in Diabetic Patients

**DOI:** 10.1371/journal.pone.0146752

**Published:** 2016-01-15

**Authors:** Donghong Jiang, Xiangqian Xiao, Tongsheng Fu, Alireza Mashaghi, Qinghuai Liu, Jiaxu Hong

**Affiliations:** 1 Department of Ophthalmology, The Second People Hospital, Taixing, China; 2 Department of Ophthalmology, People Hospital, Yangzhong, China; 3 Department of Ophthalmology and Visual Science, Eye, and ENT Hospital, Shanghai Medical College, Fudan University, Shanghai, China; 4 Department of Ophthalmology, The First Affiliated Hospital of Nanjing Medical University, Nanjing, China; 5 Massachusetts Eye and Ear Infirmary, Harvard Medical School, Boston, Massachusetts, United States of America; 6 Key Laboratory of Myopia of State Health Ministry and Key Laboratory of Visual Impairment and Restoration of Shanghai, Shangha; 7 School of Life Sciences, Xiamen University, Fujian Provincial Key Laboratory of Ophthalmology and Visual Science, Fujian, China; Wayne State University, UNITED STATES

## Abstract

**Purpose:**

Diabetes mellitus is an increasingly common systemic disease. Many diabetic patients seek cataract surgery for a better visual acuity. Unlike in the general population, the influence of cataract surgery on tear film function in diabetic patients remains elusive. The aim of this study was to evaluate the tear function in diabetic and nondiabetic patients following cataract surgery.

**Methods:**

In this prospective, interventional case series, 174 diabetic patients without dry eye syndrome (DES) and 474 age-matched nondiabetic patients as control who underwent phacoemulsification were enrolled at two different eye centers between January 2011 and January 2013. Patients were followed up at baseline and at 7 days, 1 month, and 3 months postoperatively. Ocular symptom scores (Ocular Surface Disease Index, OSDI) and tear film function including tear film stability (tear film break-up time, TBUT), corneal epithelium integrity (corneal fluorescein staining, CFS), and tear secretion (Schirmer’s I test, SIT) were evaluated.

**Results:**

In total, 83.9% of the diabetic patients (146 cases with 185 eyes) and 89.0% of the nondiabetic patients (422 cases with 463 eyes) completed all check-ups after the interventions (*P* = 0.095). The incidence of DES was 17.1% in the diabetic patients and 8.1% in the nondiabetic patients at 7 days after cataract surgery. In the diabetic patients, the incidence of DES remained 4.8% at 1 month postoperatively and decreased to zero at 3 months after surgery. No DES was diagnosed in nondiabetic patients at either the 1-month or 3-month follow-up. Compared with the baseline, the diabetic patients had worse symptom scores and lower TBUT values at 7 days and 1 month but not at 3 months postoperatively. In the nondiabetic patients, symptom scores and TBUT values had returned to preoperative levels at 1-month check-up. CFS scores and SIT values did not change significantly postoperatively in either group (*P* = 0.916 and *P* = 0.964, respectively).

**Conclusions:**

Diabetic patients undergoing cataract surgery are prone to DES. Ocular symptoms and tear film stability are transiently worsened in diabetic patients and are restored more slowly than those in nondiabetic patients.

## Introduction

As one of the most successful surgical interventions, cataract surgery involves removing the opaque lens of the eye and replacing it with an artificial lens. Although most patients achieve excellent postoperative visual acuity, tear film dysfunction associated with surgical procedures remains a major challenge[1–5]. The application of topical eye drops, impaired corneal sensitivity, and surgery-related inflammation are thought to contribute to postoperative tear film dysfunction[6–9].

Diabetes mellitus, commonly termed as diabetes, is a group of metabolic diseases with high blood sugar levels, which can cause severe vision loss or even blindness, due to ophthalmic complications such as diabetic retinopathy and cataract[10, 11]. In addition, diabetes is associated with abnormal tear quantity and quality and goblet cell loss, which play important roles in the balance of tear film function[12–14]. However, the influence of cataract surgery on tear film function in diabetic patients has not been fully investigated. To date, only one study has addressed changes in tear film and tear secretion in diabetic patients after cataract surgery. The authors reported impairment of tear secretion in diabetic patients with cataract at 6 months after phacoemulsification[15]. However, that study enrolled a very small sample (25 diabetic vs. 20 nondiabetic), and patients with prior dry eye syndrome (DES) were not excluded, an issue that may have biased the conclusions. Therefore, the aim of this study was to evaluate the tear film parameters and ocular symptoms in diabetic and nondiabetic patients undergoing cataract surgery without DES at baseline and to determine whether diabetic patients are more predisposed to the development of DES postoperatively.

## Methods

The study was conducted in accordance with the Declaration of Helsinki. The prospective study protocol was approved by the Institutional Review Board of the Second People Hospital, Taixing, Jiangsu Province, China. Written informed consent was obtained before the surgery. Between January 2011 and January 2013, patients with age-related cataract and with (*n* = 174) or without (*n* = 474) type 2 diabetes in two different eye centers were enrolled in this study. At baseline, patients who had autoimmune diseases and those who had other comorbid ocular diseases, such as DES, ocular allergies, previous ocular surgery, and ocular injury, were excluded. Patients using topical eye drops before surgery, such as preserved pressure-lowering drops, were excluded. Furthermore, patients were excluded if they had concomitant limbal relaxing incisions or developed complications during cataract surgery. After patients who were lost to follow-up were excluded, this study included 185 eyes of 146 patients (65 men, 81 women, mean age 65.4 ± 12.4 years, range from 52 years to 84 years) with type 2 diabetes and age-related cataract. The control group included 463 eyes of 422 nondiabetic patients with age-related cataract (193 men, 229 women, mean age 65.2 ± 11.9 years, range from 54 years to 91 years). Of the patients who underwent cataract surgery in both eyes, there was a period of at least 10 days between surgical procedures. The response rate was 84% in the diabetic group and 89% in the nondiabetic group, respectively (*P* = 0.095). For the analysis of the current study, only the first operated eyes were selected to exclude the possible effects of topical medication and surgical treatment.

Cataract surgery was performed by phacoemulsification through a self-sealing 3.4 to 3.8 mm clear corneal temporal incision under topical anesthesia by two of the authors (D.J. & Q.L.). A foldable posterior chamber intraocular lens was implanted in the capsular bag. Postoperatively, topical 0.3% tobramycin/0.1% dexamethasone eye drops and pranoprofen 0.1% were instilled 3 times daily for 4 weeks. Patients were examined at baseline and 7 days, 1 month, and 3 months postoperatively. Clinical examinations included a slit-lamp investigation, Ocular Surface Disease Index (OSDI) questionnaire scores, tear film break-up time (TBUT), corneal fluorescein staining (CFS), and Schirmer’s I test (SIT). Diagnosis of DES included OSDI scores higher than 15, SIT values lower than 7 mm, and TBUT values shorter than 7 s[16].

The statistical software package SPSS for Windows (version 19.0; SPSS, Inc., Chicago, IL.) was used in this study. Mean values for the diabetic and nondiabetic patients were compared with the Student *t*-test. The incidence of DES between the two groups was analyzed using the *χ*^2^ test. Analysis of variance (*ANOVA*) analysis with Bonferroni post hoc analysis was used to evaluate tear film parameters, including OSDI scores, TBUT, CFS, and SIT values at different time points. All *P* values were two-sided and considered statistically significant when the values were less than 0.05.

## Results

Data from 146 eyes of 146 diabetic patients and 422 eyes of 422 nondiabetic patients with age-related cataract were analyzed. The demographic data are shown in Table 1. No significant differences in age (*P* = 0.986), sex (*P* = 0.847), or underlying diseases (*P* = 0.574) between the two groups were identified.

**Table 1:** Clinical characteristics in diabetic and nondiabetic patients undergoing cataract surgery.

Parameters*	Diabetic (*n* = 146)	Nondiabetic (*n* = 422)
Age(years) †	65.4 ± 12.4	65.2 ± 11.9
Sex†		
Male	65 (44.5%)	193 (45.7%)
Female	81 (55.5%)	229 (54.3%)
Underlyling diseases†		
Hypertension	79 (54.1%)	213 (50.5%)
Dyslipidemia	31 (21.2%)	85 (20.1%)
Heart disease	14 (9.6%)	38 (9.0%)
Other (Parkinson, HBV, asthma,hyperthyroid, etc.)	22 (15.1%)	86 (20.4%)

* Student *t*-test or *χ*^2^ test was performed for these parameters.

† *P* > 0.05

As shown in Table 2, the incidence of DES was 17.1% in the diabetic patients and 8.1% in the nondiabetic patients at 7 days after cataract surgery (*P* = 0.004). In the diabetic patients, the incidence of DES remained 4.8% at 1 month postoperatively and decreased to zero at 3 momths after surgery. No DES was diagnosed in nondiabetic patients at either the 1 month or 3-month follow-up. Only the OSID scores and the TBUT values at 7 days (OSDI: *P* = 0.032; TBUT: *P* = 0.024) and 1 month (OSDI: *P* = 0.044; TBUT: *P* = 0.046) postoperatively showed a significant difference between the diabetic and the nondiabetic patients.

**Table 2:** Tear film parameters before and after cataract surgery in the operated eyes of diabetic and nondiabetic patients.

Parameters*	Diabetic (*n* = 146)	Non-diabetic (*n* = 422)
Dry eye syndrome incidence		
Baseline	0	0
Postoperative 7 Days†	25 (17.1%)	34 (8.1%)
Postoperative 1 Month†	7 (4.8%)	0
Postoperative 3 Months	0	0
OSDI scores		
Baseline	0.2 ± 0.1	0.2 ± 0.1
Postoperative 7 Days†	3.4 ± 0.8	2.0 ± 0.7
Postoperative 1 Month†	1.8 ± 0.7	0.7 ± 0.7
Postoperative 3 Months	0.2 ± 0.1	0.2 ± 0.1
TBUT values		
Baseline	8.0 ± 3.9	8.8 ± 3.8
Postoperative 7 Days†	4.2 ± 2.4	5.6 ± 2.7
Postoperative 1 Month†	6.2 ± 3.0	9.0 ± 3.5
Postoperative 3 months	8.3 ± 2.6	9.7 ± 2.8
SIT values		
Baseline	10.4 ± 2.7	10.3 ± 2.9
Postoperative 7 Days	9.8 ± 2.8	9.7 ± 2.4
Postoperative 1 Month	9.6 ± 2.5	10.1 ± 2.6
Postoperative 3 Months	11.4 ± 2.3	10.5 ± 3.0
CFS scores		
Baseline	1.2 ± 0.8	1.1 ± 0.8
Postoperative 7 Days	1.8 ± 0.9	1.9 ± 0.6
Postoperative 1 Month	1.5 ± 0.8	1.4 ± 0.7
Postoperative 3 Months	1.3 ± 0.5	1.2 ± 0.3

* Student *t*-test or *χ*^2^ test was performed for these parameters.

† *P* < 0.05 considered statically significant.

**Fig 1:**
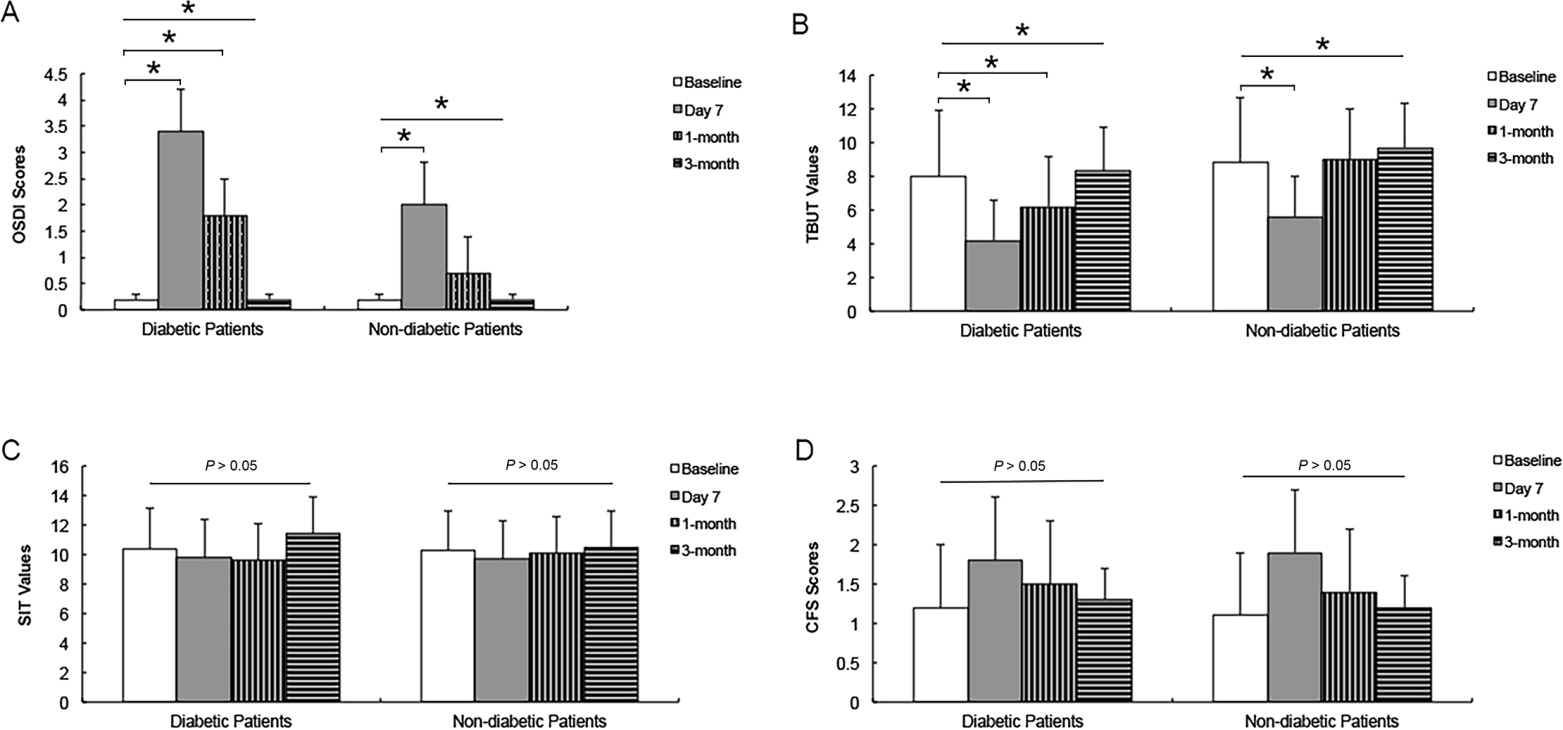
Changes in tear film parameters after cataract surgery. The ANOVA with the Bonferroni correction method was used when compared with the baseline. Higher OSDI scores (A) and lower TBUT values (B) in diabetic patients were found at 7 days and 1 month after cataract surgery, while the OSDI scores and TBUT values worsened only at 7 days postoperatively in nondiabetic patients. The postoperative CFS scores (C) and SIT values (D) of the diabetic and nondiabetic patients were similar to the baseline values. OSDI = Ocular Surface Disease Index questionnaire scores; TBUT = tear film break-up time; CFS = corneal fluorescein staining; SIT = Schirmer’s I test. * *P* < 0.05.

As shown in Fig 1, compared with the baseline, the ANOVA analysis with Bonferroni post hoc analysis showed that the diabetic patients had higher OSDI scores and lower TBUT values at 7 days and 1 month but not at 3 months postoperatively. In the nondiabetic patients, the symptom scores and TBUT values had returned to preoperative levels at the 1-month check-up. The CFS scores and SIT values did not change significantly postoperatively in either group (*P* = 0.916 and *P* = 0.964, respectively).

## Discussion

DES in normal subjects can often develop after various types of ocular surgeries such as cataract surgery[17], photorefractive keratectomy[18, 19] and laser-assisted in situ keratomileusis[20]. The aim of this study was to investigate the changes in tear film function and evaluate whether development of DES was more prevalent in diabetic than in nondiabetic patients without DES at baseline after cataract surgery. As anticipated, the incidence rate of DES was higher in diabetic patients at 7 days and 1 month postoperatively; however, at the 3-month follow-up, this difference was not significant. Similarly, we observed a statistically significant increase in ocular symptoms (OSDI scores) and decreased tear film stability (TBUT values) in the diabetic patients after cataract surgery within 1 month. The OSDI scores and decreased TBUT values in the nondiabetic patients had already returned to the baseline levels at the postoperative 1-month check-up. There were no changes in the corneal epithelium integrity (CFS scores) and tear secretion (SIT values) in either group before and after cataract surgery.

The development or aggravation of DES after cataract surgery has been well addressed in healthy subjects. It was shown that DES after phacoemulsification was commonly seen in patients with no complications. The severity of dry eye peaked 7 days after phacoemulsification, although the severity improved over time[17]. In addition, Li et al. found that the incidence of dry eye even increased dramatically after cataract surgery. They concluded that poor compliance with the use of eye drops is the main reason for this phenomenon[3]. On the other side, Ram et al. reported that although phacoemulsification can aggravate the tear film dysfunction of patients with dry eye[2], this effect seems to be present in the short-term only.[21] The recovery pattern of tear film function of non-diabetic patients in our study was in agreement with these previous reports. The exact reasons for tear film dysfunction after cataract surgery remain elusive. Oh et al. demonstrated that unlike TBUT or SIT values, the decrease in goblet cell density does not recover at 3 months after cataract surgery, which may be responsible for causing ocular discomfort and DES[22]. In addition, corneal denervation associated with surgical injuries leads to the impairment of normal blinking and tearing reflexes thus resulting in damage to the corneal epithelium[23–25]. Inflammatory cytokines released during corneal incision healing may also decrease corneal sensitivity and cause tear film instability[26, 27].

Diabetes has been widely reported as a leading systemic risk factor for DES[28, 29]. Corneal neuropathy and microvascular complications associated with diabetes could significantly decrease tear film function and corneal sensitivity[29–31]. Yet tear film changes in diabetic patients after cataract surgery remains largely unexplored. In an animal model, Gemensky-Metzler et al. documented that DES is almost twice as prevalent during the first 2 weeks after cataract surgery in diabetic compared to nondiabetic dogs[32]. In a small case series, Liu et al. observed that tear secretion was decreased in diabetic patients after phacoemulsification, which resulted in worsening dry eye symptoms and damage to the corneal epithelium integrity[15]. However, at baseline, the diabetic patients in that study already had decreased TBUT and SIT values, meaning that DES may have existed in this population before surgery. To exclude the effect of baseline DES on tear film changes in diabetic patients after cataract surgery, we used strict inclusion criteria to enroll patients with normal tear film function. As anticipated, the DES incidence was significantly higher in diabetic patients at 7 days and at 1 month after cataract surgery. The OSDI scores and TBUT values seem to recover more slowly than in nondiabetic patients. The CFS scores and SIT values, however, did not change significantly postoperatively in either group. Our results are consistent with the results of Kasetsuwan et al. who found that DES developed immediately after phacoemulsification and its severity peaked on day 7 but rapidly improved within 30 days postoperatively[17]. Conversely, Liu et al. found that SIT values were reduced in diabetic cataract patients after phacoemulsification, meaning that tear production was even weakened after surgery. [15] In addition, although Liu et al. reported that diabetic patients showed significantly increased CFS scores after cataract surgery,[15] Han et al. did not detect any change in CFS scores when both diabetic and non-diabetic patients were included[5]. Our study exclusively focused on diabetic patients without DES at baseline and we found that a fraction of the population develops DES within a short-term after cataract surgery. When averaging these diabetic patients, we also found an increase in the CFS scores (Fig 1D), which was similar to the report of Liu et al., but this increase did not reach statistical significance. The emergence of postoperative DES could either due to inherent susceptibility of diabetic patients or due to the damage caused by surgeries. It seems that the self-limited damage to corneal epithelium in the current study was mostly induced by the surgical procedure, including the use of ultrasound, the application of topical anaesthesia eye drops, the corneal incision and so on[24, 33]. Because of different sampling strategies, different tear film function parameters in diabetic patients, and different grade systems for DES-related symptoms and signs, a direct comparison with previous reports may not be available. Further studies from other ethnic populations are still warranted to validate our findings.

Several limitations in this study should be noted. First, Meibomian gland function was not fully studied, because this test requires us to depress the lid, which may make the corneal incision reopen. Second, information on blood sugar levels and duration of disease was lacking. Some diabetic patients were undiagnosed or untreated until the surgery[34]. Third, corneal sensitivity was not measured in our study due to financial limitations. However, it has been reported that after corneal surgery, neural growth factor is released to repair the corneal nerve, which needs about 1 month to complete[25].

In conclusion, our results provide important information showing that diabetic patients undergoing cataract surgery are prone to DES. Ocular symptoms and tear film stability are only transiently worsened in diabetic patients and are restored more slowly than in nondiabetic patients. Based on our findings, careful monitoring of tear film function and the extended use of artificial tear supplements after cataract surgery may be warranted for diabetic patients.

## Abbreviations

DES: dry eye syndrome
OSDI: Ocular Surface Disease Index
TBUT: tear film break-up time
CFS: corneal fluorescein staining
SIT: Schirmer’s I test
